# Bayesian structured additive regression modeling of epidemic data: application to cholera

**DOI:** 10.1186/1471-2288-12-118

**Published:** 2012-08-06

**Authors:** Frank B Osei, Alfred A Duker, Alfred Stein

**Affiliations:** 1 Faculty of Public Health and Allied Sciences, Catholic University College of Ghana, Sunyani/Fiapre, Ghana; 2 Department of Geomatic Engineering, Kwame Nkrumah University of Science and Technology, Kumasi, Ghana; 3 Faculty of Geo-Information Science and Earth Observation-ITC, Twente University, Enschede, Netherlands

**Keywords:** Bayesian, Cholera, Cholera reservoir, Refuse dumps, Slums

## Abstract

**Background:**

A significant interest in spatial epidemiology lies in identifying associated risk factors which enhances the risk of infection. Most studies, however, make no, or limited use of the spatial structure of the data, as well as possible nonlinear effects of the risk factors.

**Methods:**

We develop a Bayesian Structured Additive Regression model for cholera epidemic data. Model estimation and inference is based on fully Bayesian approach via Markov Chain Monte Carlo (MCMC) simulations. The model is applied to cholera epidemic data in the Kumasi Metropolis, Ghana. Proximity to refuse dumps, density of refuse dumps, and proximity to potential cholera reservoirs were modeled as continuous functions; presence of slum settlers and population density were modeled as fixed effects, whereas spatial references to the communities were modeled as structured and unstructured spatial effects.

**Results:**

We observe that the risk of cholera is associated with slum settlements and high population density. The risk of cholera is equal and lower for communities with fewer refuse dumps, but variable and higher for communities with more refuse dumps. The risk is also lower for communities distant from refuse dumps and potential cholera reservoirs. The results also indicate distinct spatial variation in the risk of cholera infection.

**Conclusion:**

The study highlights the usefulness of Bayesian semi-parametric regression model analyzing public health data. These findings could serve as novel information to help health planners and policy makers in making effective decisions to control or prevent cholera epidemics.

## Background

A significant interest in understanding the epidemiology of diseases lies in identifying associated risk factors which enhance the risk of infection, the so called *ecological studies*[1,2]. Most of these ecological studies, however, make no, or limited use of the spatial structure of the data, neither do they consider possible nonlinear effects of the risk factors. Thus, most studies use standard statistical methods such as the classical and generalized linear models that ignore methodological difficulties that arise from the nature of the data. Ali *et al.*[3,4] have used logistic, simple and multiple linear regression models to study the spatial epidemiology of cholera in an endemic area of Bangladesh. Other ecological studies of cholera that have utilized standard statistical methods include Ackers *et al.*[5], Mugoya *et al.*[6] and Sasaki *et al.*[7]. These methods when applied to spatially distributed data present severe problems with estimating small area spatial effects, and simultaneously adjusting for other risk factors, in particular if such effects are nonlinear. If standard statistical methods are used to analyze spatially correlated data, the standard error of the covariate parameters is underestimated and thus the statistical significance is overestimated [8].

Generalized additive models (GAM) provide a powerful class of models for modeling nonlinear effects of continuous covariates in regression models with non-Gaussian responses. Structured Additive Regression (STAR) models are extensions of GAM models that allow one to incorporate small area spatial effects, nonlinear effects of risk factors, and the usual linear or fixed effects in a joint model [9]. This study applies a STAR modeling approach to develop a multivariate explanatory model for cholera.

Cholera outbreak is enhanced by several environmental and/or socioeconomic risk factors once introduced in a population. Ali *et al.*[3,4] identified proximity to surface water, high population density, and low educational status as the important risk factors of cholera in an endemic area of Bangladesh. Borroto and Martinez-Piedra [10] identified poverty, low urbanization, and proximity to coastal areas as the important geographic risk factors of cholera in Mexico. Sanitation is an important environmental risk factor that predisposes inhabitants to cholera infection. Previous ecological studies have used spatial regression models to explore the dependency of cholera on some local measures of sanitation [11,12]. No attempt, however, has been made to combine all the identified measures of sanitation, including spatial effects, into a single multivariate model to examine their joint effects on cholera. In this study, we exploit the joint effects of three main spatial measures of sanitation identified from previous studies [11,12]. These are density of refuse dumps, proximity to refuse dumps and proximity to potential cholera reservoirs. Other risk factors used in this study include livelihood at slummy and squatter environments [13], and population density [3,4,14,15]. Livelihood at slummy and squatter environments increase the risk of cholera infection, whereas high population density stresses existing sanitation systems, thus putting people at increased risk of cholera.

This study incorporates the effects of nonlinear risk factors and the usual fixed effects of some risk factors, while accounting for both structured and non structured spatial effects. A STAR model of this type has been termed *geoadditive* model [16,17]. The increasing availability of disease and environmental data necessitate the development of such models to obtain valid and realistic statistical inferences that adequately describe the variation of the disease. Proximity to dumps, density of dumps, and proximity to potential cholera reservoirs are modeled as smooth continuous functions, whereas presence of slum settlers and population density are modeled as fixed effects, and spatial references to the communities are modeled as structured and unstructured spatial effects. We use a fully Bayesian estimation based on Markov Chain Monte Carlo (MCMC) simulations using simple Gibbs sampling updates. Making inferences based on a fully Bayesian approach is preferred because the functionals of the posterior can be computed without relying on large Gaussian justifications, thereby quantifying the uncertainty in the parameters [18].

## Methods

### Study area and cholera data

**Figure 1:**
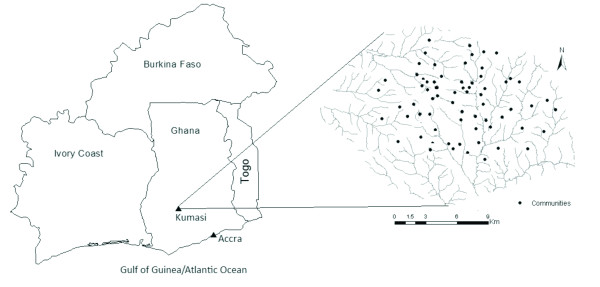
**Map of Ghana and neighboring countries (left), and Kumasi (right).** Dots indicate the centroids of communities.

This study is based on the 2005 cholera outbreak in Kumasi Metropolis, Ghana. Kumasi Metropolis is completely urban and the most populous city in Ashanti Region. It is located at the intersection of latitude 6.04°N and longitude 1.28°W, covering an area of approximately 220 km^2^ (See Figure 1). Kumasi has a population of approximately 1.2 million. Surveillance and reporting of the disease before 2005 has been ineffective, and hence the existing data before 2005 have little or no spatial information. However, with intensified surveillance and reporting systems during an outbreak in 2005, disease cases in Kumasi are available at community level spatial units. This makes the Kumasi area suitable for such a study. During the outbreak in 2005, cholera incidence rates ranged from 0.47 to 31.92 per 10,000 people (*mean* = 10.21, *standard deviation* = 6.84).

The topographic map of the metropolis and the *n = 68* communities where cholera records are available was digitized. Cholera data for each community was extracted from disease records of the Kumasi Metropolitan Disease Control Unit (DCU). We accessed such data based on special permissions given by the Kumasi DCU. The centroids of the communities were used as the spatial references of cholera cases since residential addresses were not recorded during the outbreak. The denominator (population data) for computing community-specific cholera rates was obtained from the 2000 Population and Housing Census of Ghana [19].

### Model specification

For each community *i*, i=1,…,N of population *P*_*i*_, the observed number of cholera cases $Chol_{\left(O\right)i}$ is assumed to be a realization of random variable that follows independent Poisson distribution with intensity$Chol_{\left(E\right)i}\cdot Chol_{\left(R\right)i}$; thus: $Chol_{\left(O\right)i}|Chol_{\left(R\right)i}∼\text{Poisson}\left(Chol_{\left(E\right)i}\cdot Chol_{\left(R\right)i}\right)$, where $Chol_{\left(E\right)i}$ is the expected number of cholera cases and $Chol_{\left(R\right)i}$is the relative risk of cholera infection. A common practice is to estimate $Chol_{\left(E\right)i}$ as ${Chol}_{\left(R\right)}\cdot P_{i}$, where ${Chol}_{\left(R\right)}$ is the overall risk of cholera infection within the study population obtained as a weighted average of the community-specific rates, each weighted by their share in the overall population; thus:

$${Chol}_{\left(R\right)}=\sum_{i=1}^{N}\frac{Chol_{\left(O\right)i}}{P_{i}}\times \frac{P_{i}}{\sum_{i=1}^{N}P_{i}}\text{.}$$

For ease of interpretation, we use the relative risk (also called excess risk) as the reference benchmark to estimate the risk of cholera infection. We consider the triple $(Chol_{(R)i},x_{i},w_{i})\text{,}\;i=1,\ldots ,N$ where $Chol_{\left(R\right)i}$ is the relative risk of cholera infection in community *i*. The vector $x_{i}={\left(x_{i1},\ldots ,x_{\mathrm{ip}}\right)}^{\prime }$ contains the *p* continuous covariates and $w_{i}={\left(w_{i1},\ldots ,w_{\mathrm{ir}}\right)}^{\prime }$ is a vector of *r* categorical covariates. In our study, *p =* 3 and *r =* 2*.* The study assumes that the response variable *Chol*_(*R*)_ is Gaussian distributed, i.e.$Chol_{\left(R\right)i}{|\eta }_{i},\sigma^{2}\sim N(\eta_{i},\sigma^{2})$, with an unknown mean *η*_*i*_ which can be expressed in the form:

(1) $$\eta_{i}={x^{\prime }}_{i}\beta +{w^{\prime }}_{i}\gamma \text{.}$$

Here, *β* is a *p*-dimensional vector of unknown regression coefficients for the continuous covariates *x*_*i*_, and *γ* is a *r*-dimensional vector of unknown regression coefficients for the categorical covariates *w*_*i*_.

In order to account for both the nonlinear effects of the continuous covariates and the spatial dependence of the data, a *geoadditive* modeling approach is required [16]. The *geoadditive* model replaces the strictly linear predictor by a more flexible semi-parametric predictor as:

(2) $$\eta_{i}=f_{1}\left(x_{i,1}\right)+\ldots +f_{p}\left(x_{i,p}\right)+f_{\mathrm{spat}}\left(s_{i}\right)+{w^{\prime }}_{i}\gamma \text{.}$$

Here, $f_{1}\left(x\right),\ldots ,f_{p}\left(x\right)$ are nonlinear smooth functions of the continuous covariates $x_{i,1},\ldots ,x_{i,p}$ and $f_{\mathrm{spat}}\left(s_{i}\right)$ is a function that accounts for spatial effects at each community $s_{i}\in \left\{1,\ldots ,S\right\}$. Spatial effect is usually a surrogate of unobserved influential factors, some of which may have a strong spatial structure and others may be present only locally (unstructured). To distinguishing between the two kinds of influential factors$f_{\mathrm{spat}}\left(s\right)$is split up into spatially correlated (smooth) part $f_{\mathrm{str}}\left(s\right)$and spatially uncorrelated (unsmooth) part$f_{\mathrm{unstr}}\left(s\right)$, i.e. $f_{\mathrm{spat}}\left(s\right)=f_{\mathrm{str}}\left(s\right)+f_{\mathrm{unstr}}\left(s\right)$.

The final *geoadditive* model is then expressed as:

(3) $$\begin{array}{l} \eta_{i}=f_{1}\left(x_{i,1}\right)+\ldots +f_{p}\left(x_{i,p}\right)+f_{\text{str}}\left(s_{i}\right)\\ \;+f_{\text{unstr}}\left(s_{i}\right)+{w^{\prime }}_{i}\gamma \text{.} \end{array}$$

This model contains *p* + 2 functions and *r* fixed parameters to be estimated.

### Prior distributions for covariates

A fully Bayesian approach for modeling and inferences requires prior assumptions for the unknown functions $f_{j}\left(x\right)\text{,}\;f_{\mathrm{unstr}}\left(s\right),f_{\mathrm{str}}\left(s\right)$ and the fixed effect regression parameter *γ*. For *γ*, we assume an independent diffuse prior$p\left(\gamma \right)\propto const$ due to the absence of any prior knowledge. A possible alternative choice is a weak informative multivariate Gaussian distribution.

For the continuous functions$f_{j}\left(x\right),j=1,\ldots ,p$, we choose the Bayesian P(enalized)-splines [20,21]. This approach assumes that an unknown smooth function *f*_*j*_ of a covariate *x*_*j*_ can be approximated by a polynomial spline of degree *l* defined on a set of equally spaced knots $x_{j}^{\text{min}}=\zeta_{j,0}<\zeta_{j,1}<\cdots <\zeta_{j,s-1}<\zeta_{j,s}=x_{j}^{\text{max}}$within the domain of *x*_*j*_. Such a spline can be written in terms of a linear combination of d=s+l basis functions *B*_*m*_, i.e.

(4) $$f_{j}\left(x_{j}\right)=\sum_{m=1}^{d}\xi_{j,m}\cdot B_{m}\left(x_{j}\right)\text{.}$$

The B-splines form a local basis since the functions *B*_*m*_ are only positive within an area spanned by *l* + 2 knots. This property is essential for the construction of the smoothness penalty for P-splines. The estimation of *f*_*j*_ (*x*_j_) is thus reduced to the estimation of the vector of unknown regression coefficients $\xi_{j}={\left(\xi_{j,1},\ldots ,\xi_{j,m}\right)}^{\prime }$ from the data. An essential factor in the estimation procedure is the choice of the number of knots. We chose a moderately large number of equally spaced knots (20), as suggested by Eilers and Marx [20] to ensure enough flexibility to capture the variability of the data. In the Bayesian approach, penalized splines are introduced by replacing the difference penalties with their stochastic analogues, i.e., first or second order random walk priors for the regression coefficients. A first order random walk prior for equidistant knots is given by:

(5) $$\xi_{j,m}=\xi_{j,m-1}+u_{j,m},m=2,\ldots ,d\text{,}$$

and a second order random walk for equidistant knots by:

(6) $$\xi_{j,m}=2\xi_{j,m-1}-\xi_{j,m-2}+u_{j,m},m=3,\ldots ,d\text{,}$$

where $u_{j,m}\sim N\left(0,\tau_{j}^{2}\right)$are Gaussian errors. Diffuse priors$\xi_{j,1}\propto const$, or $\xi_{j,1}$ and$\xi_{j,2}\propto const$, are chosen as initial values, respectively. The joint distribution of the regression parameters$\xi_{j,m}$for a first order random walk is defined as:

(7) $$\xi_{j,m}|\xi_{j,m-1}\sim N\left(\xi_{j,m-1},\tau_{j}^{2}\right)\text{,}$$

and a second order random walk is defined as:

(8) $$\xi_{j,m}|\xi_{j,m-1},\xi_{j,m-2}\sim N\left(2\xi_{j,m-1}-\xi_{j,m-2},\tau_{j}^{2}\right)\text{.}$$

The first order random walk induces a constant trend for the conditional expectation of $\xi_{j,m}$ given $\xi_{j,m-1}$and a second order random walk results in linear trend depending on the two previous values $\xi_{j,m-1}$and $\xi_{j,m-2}$. The joint distribution of the regression parameters $\xi_{j}={\left(\xi_{j,1},\ldots ,\xi_{j,m}\right)}^{\prime }$is computed as a product of the conditional densities defined by the random walk priors. The general form of the prior for *ξ*_*j*_ is a multivariate Gaussian distribution with density:

(9) $$p(\xi_{j}|\tau_{j}^{2})\propto \exp\left(-\frac{{\xi^{\prime }}_{j}K_{j}\xi_{j}}{2\tau_{j}^{2}}\right)\text{,}$$

where the precision matrix *K*_*j*_ acts as a penalty matrix that shrinks parameters towards zero, or penalizes too abrupt jumps between neighboring parameters. Since the penalty matrix *K*_*j*_ is rank deficient, i.e.$k_{j}=\text{rank}\left(K_{j}\right)<\dim\left(\xi_{j}\right)=d_{j}$, it follows that the prior for $\xi_{j}|\tau_{j}^{2}$ is partially improper with Gaussian prior $\xi_{j}|\tau_{j}^{2}\propto N\left(0;\tau_{j}^{2}K_{j}^{-}\right)$, where $K_{j}^{-}$is a generalized inverse of *K*_*j*_. The tradeoff between flexibility and smoothness is controlled by the variance parameter $\tau_{j}^{2}$. A large variance corresponds with a rough estimated function, and vice versa.

### Spatial components

We use the nearest neighbor Gaussian Markov random field model which is common in spatial statistics to express prior knowledge of the structured spatial effects. Suppose $s\in \left\{1,\ldots ,S\right\}$represent the locations of connected communities, then the locally dependent prior probability spatial structure can be specified as:

(10) $$f_{\mathrm{str}}\left(s\right)|f_{\mathrm{str}}\left(s^{\prime }\right),s^{\prime }\neq s,\tau_{\mathrm{str}}^{2}\sim N\left(\frac{1}{N_{s}}\sum_{s^{\prime }\in \partial s}f_{\mathrm{str}}\left(s^{\prime }\right),\frac{\tau_{\mathrm{str}}^{2}}{N_{s}}\right)\text{,}$$

where *N*_*s*_ is the number of adjacent spatial units and $s^{\prime }\in \partial s$denotes that spatial unit *s’* is a neighbor of spatial unit *s*. Thus, the conditional mean of *f*_*str*_ (*s*) is an unweighted average of the function evaluations of neighboring spatial units. Since only the centroids of communities (point data) are available, we assume the effect of spatial interaction is dependent on distance between the centroids of pair of communities. To ensure equal number of neighbors for each community we chose a neighborhood structure based on the *k*th nearest neighbor method (where *k* is the number of neighbors). This approach results in an asymmetric neighborhood matrix; therefore, false symmetry was imposed to ensure a symmetrical neighborhood structure. Like the continuous functions *f*_*j*_, the tradeoff between flexibility and smoothness is controlled by the variance parameter$\tau_{\mathrm{str}}^{2}$.

For the unstructured spatial effects, we assume that the parameters *f*_*unstr*_ (*s*) are *i.i.d*. Gaussian:

(11) $$f_{\mathrm{unstr}}\left(s\right){|\tau }_{\mathrm{unstr}}^{2}∼N\left(0;\tau_{\mathrm{unstr}}^{2}\right)\text{.}$$

Hyperpriors for the variance or smoothness parameters $\tau_{j}^{2}\text{,}$j=1,…,p,*str*, *unstr*, are considered as unknown. Therefore, highly dispersed, but proper, inverse Gamma distributions $p\left(\tau_{j}^{2}\right)∼IG\left(a_{j},b_{j}\right)$with known hyper-parameters *α*_*j*_ and *b*_*j*_ are assigned in the second stage of the hierarchy. The corresponding probability density function is expressed as:

(12) $$p\left(\tau_{j}^{2}\right)\propto {\left(\tau_{j}^{2}\right)}^{-a_{j}-1}\exp\left(-\frac{b_{j}}{\tau_{j}^{2}}\right)\text{.}$$

In this study, we use the standard option hyper-parameters proposed by Farhmeir *et al.*[18]: *IG* (*a* = *b* = 0.001).

### Bayesian inference

Bayesian inference stems from the posterior distribution, that is, the conditional distribution of the model parameters given the observed data$p\left(\theta |Chol_{\left(R\right)}\right)$, where *θ* denotes the vector of all model parameters, *Chol*_(*R*)_ the data vector, *p* (.) represents the probability density function. In this study, we use a fully Bayesian inference based on analysis of posterior distribution of the model parameters by drawing random samples via MCMC simulation techniques. The probability density function of the posterior distribution is expressed as:

(13) $$p(\theta |Chol)\propto \prod_{i=1}^{n}L(Chol_{(R)i},\eta_{i})\times \prod_{j=1}^{p}[p(\xi_{j}|\tau_{j}^{2})p(\tau_{j}^{2})]\times p(f_{\text{str}}|\tau_{\text{str}}^{2})p(f_{\text{unstr}}|\tau_{\text{unstr}}^{2})\times \prod_{j=1}^{r}p\left(\gamma_{j}\right)p\left(\sigma^{2}\right)\text{,}$$

where *L* (.) is the likelihood function. The full conditional for the variance components $\tau_{j}^{2},j=1,\ldots ,p\text{,}$*str*, *unstr*, and *σ*^2^ are inverse Gamma distributions. The full conditional for the fixed parameters *γ*, the unknown parameter vector$\xi_{1},\ldots ,\xi_{p}$, as well as $f_{\mathrm{str}}\left(s\right)\text{,}$$f_{\mathrm{unstr}}\left(s\right)$ are multivariate Gaussian. Gibbs sampler was employed for MCMC simulations, drawing successively from the full conditionals for the variance components and the unknown parameters. Cholesky decompositions for band matrices were used to efficiently draw random samples from the full conditional [22,23].

### Model implementation

The continuous covariates used in this study are *proximity to refuse dumps d*_*dumps*_, *density of refuse dumps ρ*_*dump*_, and *proximity to potential cholera reservoirs d*_*reser*_. These variables are extracted on per community basis via a Geographic Information System (GIS). Details of the approaches for the calculation of these variables can be found in Osei and Duker [11] and Osei *et al.*[12]. The spatial locations of the communities are used to model the spatial effects. In the Kumasi area no administrative boundaries are present separating the communities. For ease of visualization and interpretation, the centroids of the communities are converted to Thiessen polygons whose boundaries define the area that is closest to each centroid relative to all other centroids.

In addition, two binary categorical covariates are used; *presence of slum settlers in a community*$\varsigma_{\mathrm{slum}}$and *population density ρ*_*pop*_. For communities within which slum settlers dwell, $\varsigma_{\mathrm{slum}}$ =1, otherwise $\varsigma_{\mathrm{slum}}$=0. Since the boundaries of the various communities do not exist the population density could not be quantified as continuous variable. Therefore, we categorized the population density as moderately populated $\rho_{\mathrm{pop}}=0$ and densely populated $\rho_{\mathrm{pop}}=1$. We analyze the following set of models.

$$\begin{array}{l} \text{Model}\;1:\eta_{i}\\ \;={\rho^{\prime }}_{\text{dump}}\beta_{1}+{d^{\prime }}_{\text{dump}}\beta_{2}+{d^{\prime }}_{\text{reser}}\beta_{3}+{\rho^{\prime }}_{\text{pop}}\gamma_{1}\\ \;+{\varsigma^{\prime }}_{\text{slum}}\gamma_{2} \end{array}$$

$$\begin{array}{l} \text{Model}\;2:\eta_{i}\\ \;=f_{1}\left(\rho_{\text{dump}}\right)+f_{2}\left(d_{\text{dump}}\right)+f_{3}\left(d_{\text{reser}}\right)\\ \;+{\rho^{\prime }}_{\text{pop}}\gamma_{1}+{\varsigma^{\prime }}_{\text{slum}}\gamma_{2} \end{array}$$

$$\begin{array}{l} \text{Model}\;3:\eta_{i}\\ \;=f_{1}\left(\rho_{\text{dump}}\right)+f_{2}\left(d_{\text{dump}}\right)+f_{3}\left(d_{\text{reser}}\right)+f_{\text{str}}\left(s\right)\\ \;+f_{\text{unstr}}\left(s\right)+{\rho^{\prime }}_{\text{pop}}\gamma_{1}+{\varsigma^{\prime }}_{\text{slum}}\gamma_{2} \end{array}$$

Model 1 is a strictly linear regression that assumes a linear effect of the categorical and continuous covariates. Model 2 is an additive model which assumes nonlinear functions for the continuous covariates and linear effects of the categorical covariates. Model 3 is a geoadditive model, which is an extension of Model 2 that incorporates both structured and unstructured spatial effects.

The models were implemented in the public domain software BayesX ver 2.0 [24,25]. We used a total number of 40,000 MCMC iterations and 10,000 number of burn in samples. Since, in general, these random numbers are correlated, only every 20^th^ sampled parameter of the Markov chain were stored. This yielded 2,000 samples for parameter estimation. Convergence checks of the MCMC algorithms were based on autocorrelations and the sampling paths.

We compared the strictly linear models with the additive models and the geoadditive models using the Deviance Information Criterion (*DIC*) values [26]. *DIC* is a Bayesian tool for model checking and comparison, where the model with the smallest *DIC* is preferred. The *DIC* is given by$DIC=\bar{D}+p_{D}$, where $\bar{D}$ is the posterior mean of the deviance, which is a measure of goodness of fit, and *p*_*D*_ is the effective number of parameters, which is a measure of model complexity and penalizes over-fitting.

## Results

### Model selection

Model assessment and selection was based on the computed values for the goodness of fit (see Table 1). Models with a smaller *DIC* value are preferred. Again, models with differences in *DIC* of less than 3 cannot be distinguished, while those between 3 and 7 can be weakly differentiated [27]. Comparing goodness of fit of models, Model 3 is the preferred model. Although the extension of the basic model (Model1) to an additive model (Model 2) is an improvement; this improvement is indistinguishable (*DIC* = 43.25 in Model 1 versus *DIC* = 41.30 in Model 2, IC=1.95). The extension of Model 2 to include structured and unstructured spatial effects in Model3 significantly improved the model (*DIC* = 20.07 in Model 3 versus *DIC* = 41.30 in Model 2, IC=21.23). Therefore, subsequent analysis and discussions are based on the results of Model 3.

**Table 1:** **Comparison of model fit using Deviance Information Criterion (*DIC*)**

**Model Fit**	**Model 1**	**Model 2**	**Model 3**
$\bar{D}$	37.40	32.35	10.64
_*pD*_	5.85	8.95	9.43
_*DIC*_	43.25	41.30	20.07
_Δ*DIC*_^§^	23.18	21.23	Reference

^§^Difference of Model 3 against Models 1&2.

### Fixed and nonlinear effects of covariates

**Figure 2:**
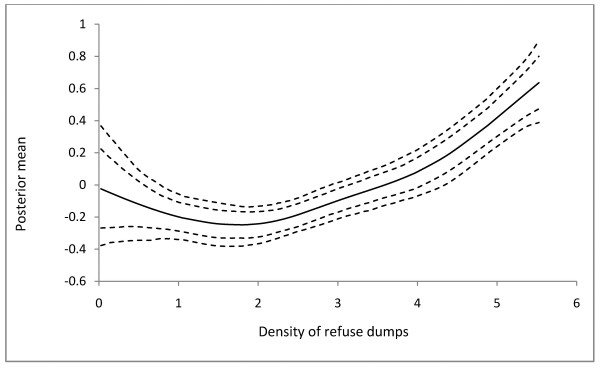
**The estimated nonlinear effects of cholera risk on of proximity to refuse dumps in Kumasi.** The posterior mean together with the 80% and 90% credible intervals are shown.

**Figure 3:**
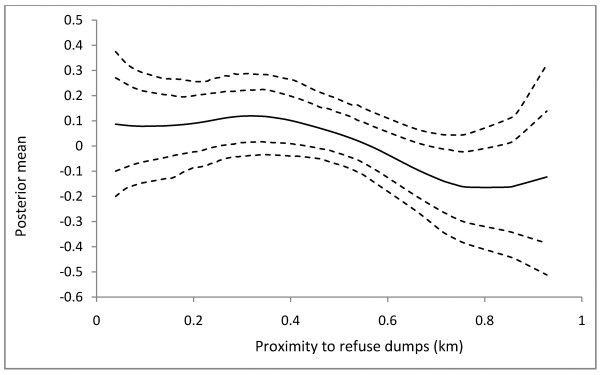
**The estimated nonlinear effects of cholera risk on dumps density in Kumasi.** The posterior mean together with the 80% and 90% credible intervals are shown.

**Figure 4:**
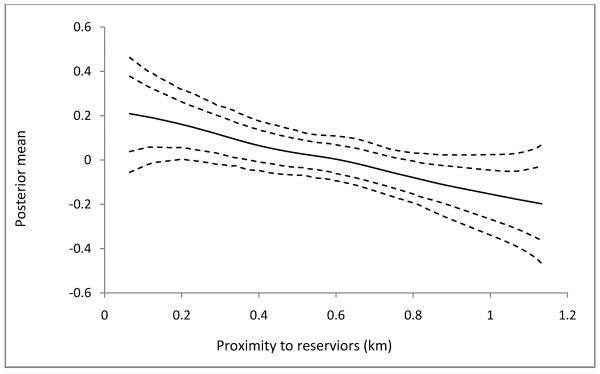
**The estimated nonlinear effects of cholera risk on proximity to potential cholera reservoirs in Kumasi.** The posterior mean together with the 80% and 90% credible intervals are shown.

The purpose of Model 1 has been to investigate the appropriateness of including nonlinear effects in disease modeling. In Model 1, the continuous covariates *ρ*_*dump*_ and *d*_*reser*_ are observed to have no significant effect on *Chol*_(*R*)_ which would have led to an erroneous rejection of the significance of their effect (Table 2). In Model 3, the effects of the categorical covariates are assumed fixed are estimated jointly with the continuous and spatial covariates. The posterior means and the corresponding 90% credible intervals of the fixed effect parameters are shown in Table 3. The risk of cholera infection is observed to be associated with high population density and livelihood at slummy environments. Moderate difference occurs between the risk of infection in populous communities and the risk of infection in slummy. Thus the effect of *ρ*_*pop*_ on *Chol*_(*R*)_ is 0.32 (0.20 - 0.44) and the effect of *ς*_*slum*_ on *Chol*_(*R*)_ is 0.28 (0.16 - 0.40). The nonlinear effects of *ρ*_*dump*_, *d*_*dump*_, and *d*_*reser*_ are shown in Figures 2, 3, and 4, respectively. The relationship between *Chol*_(*R*)_ and *ρ*_*dump*_ is nonlinear, with an expected increasing risk (Figure 2), preceded by approximate equal risk up to$\rho_{\mathrm{dump}}=1.8$. In other words, the risk of cholera infection is equal and lower for communities with fewer refuse dumps, but increases with increasing refuse dumps from$\rho_{\mathrm{dump}}=1.8$. For *d*_*dump*_, the risk of infection remains constant up to approximately 500 m, and then deviates from linearity with a general decreasing trend (Figure 3). The effect of *d*_*reser*_ is almost linear, with the posterior mean decreasing with increasing distance (Figure 4).

**Table 2:** Estimates of fixed effect parameters based on the linear Model 1

**Variable**	**Mean**	**Std. error**	**10%**	**90%**
*constant*	0.444*	0.213	0.171	0.718
$\varsigma_{\text{slum}},\gamma_{2}$	0.267*	0.098	0.141	0.393
$\rho_{\text{pop}},\gamma_{1}$	0.344*	0.089	0.230	0.457
$\rho_{\text{dump}},\beta_{1}$	0.156*	0.039	0.107	0.206
$d_{\text{dump}},\beta_{2}$	4.99E-05	7.19E-05	−4.40E-05	0.00014
$d_{\text{reser}},\beta_{3}$	−6.54E-05	6.42E-05	−1.44 E-04	1.63E-05

* Significance at *p <0 .01*.

**Table 3:** Estimates of posterior mean and 90% credible intervals for the fixed effects for Model 3

**Variable**	**Mean**	**Std. error**	**10%**	**90%**
Constant	0.73*	0.081	0.63	0.83
$\varsigma_{\text{slum}},\gamma_{2}$	0.28*	0.095	0.16	0.40
$\rho_{\text{pop}},\gamma_{1}$	0.32*	0.092	0.20	0.44

*Significance at *p <0 .01*.

### Spatial effects

**Figure 5:**
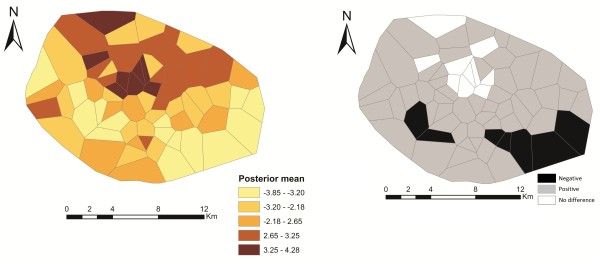
**Spatial distribution of the posterior means of the total spatial effects on cholera risk (left), and posterior probabilities at nominal level of 80% (right).** Black denotes areas with strictly negative credible intervals; white denotes areas with strictly positive credible intervals, whereas grey shows areas of no significant difference.

**Figure 6:**
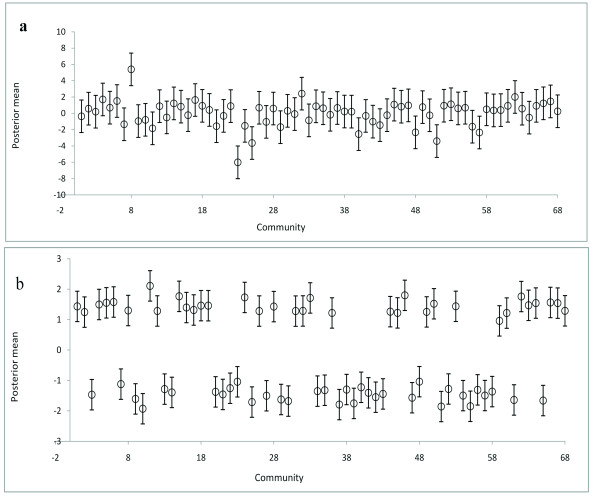
**Caterpillar plots of the posterior means of the structured (a**)** and unstructured **(**b**)** spatial effects of the risk of cholera infection, with 90% error bars.**

Figure 5 shows the estimated total spatial effects (left) and the corresponding 80% (credible interval) posterior probability map (right) of cholera risk. Areas shaded black show strictly negative credible intervals, while white areas depict strictly positive credible intervals, and grey indicate areas of non-significant spatial effects. There is evidence of significant clustering of cholera, with higher cholera risk occurring at the central part, and a lower risk occurring at the south-eastern part (the periphery) of Kumasi (Figure 5). The unstructured spatial effects are dominant over the structured spatial effects. This is shown by the higher ratio of variance components $\phi_{\text{unstr}}=\tau_{\text{unstr}}^{2}/\left(\tau_{\text{str}}^{2}+\tau_{\text{unstr}}^{2}\right)=0.64$ (Table 4). The lesser variations in the caterpillar plots of Figure 6a compared with Figure 6b also confirms that the unstructured spatial effects are dominant over the structured spatial effects.

**Table 4:** Summary of the sensitivity analysis of the choice of hyper-parameters for Model 3

	***a*** **= 0.001**	***a*** **= 0.01**	***a*** **= 0.5**	***a*** **= 1**
	***b*** **= 0.001**	***b*** **= 0.01**	***b*** **= 0.0005**	***b*** **= 0.005**
*Spatial effects*^‡^				
$f_{\text{str}}\left(s\right)$, $\tau_{\text{str}}^{2}$	0.02	0.028	0.004	0.004
	(0.0005 - 0.0.06)	(0.003 - 0.07)	(0.00009 - 0.01)	(0.0006 - 0.0009)
$f_{\text{unstr}}\left(s\right)$, $\tau_{\text{unstr}}^{2}$	0.02	0.031	0.007	0.0071
	(0.0009 - 0.0.057)	(0.005 - 0.056)	(0.0001 - 0.028)	(0.0006 - 0.019)
*Smooth functions*^§^				
$f_{1}\left(\rho_{\text{dump}}\right)$,$\tau_{1}^{2}$	0.003	0.006	0.0014	0.002
	(0.0005 - 0.006)	(0.002 - 0.013)	(0.0002 - 0.003)	(0.0006 - 0.004)
$f_{2}\left(d_{\mathrm{dump}}\right)$,$\tau_{2}^{2}$	0.003	0.0078	0.0007	0.002
	(0.0002 - 0.0058)	(0.002 - 0.017)	(0.00008 - 0.0015)	(0.0004 - 0.004)
	0.001	0.004	0.0004	0.001
$f_{3}\left(d_{\mathrm{reser}}\right)$,$\tau_{3}^{2}$	(0.0002 - 0.0024)	(0.001 - 0.009)	(0.00006 - 0.0007)	(0.0004 - 0.003)

‡ Variance components and 90% credible intervals for the spatially structured and unstructured effects; §variance components and 90% credible intervals for the nonlinear smooth functions.

### Sensitivity analyses

Since the regression parameters depend on the choice of hyper-parameters, we rerun the MCMC simulations, using Model 3 for simplicity, to investigate the sensitivity of our results to different choices of hyper-parameters. In particular, the following alternatives of priors have been investigated: *IG* (*a* = 0.01, *b* = 0.01), *IG* (*a* = 0.5, *b* = 0.0005) and *IG* (*a* = 1, *b* = 0.005). The first alternative and the standard option *IG* (*a* = 0.001, *b* = 0.001) are commonly used choices for the variances of random effects. The second and third alternatives are suggested by Kelsall and Wakefield [28] and Besag and Kooperberg [27], respectively. Results of the sensitivity analysis on the choice of hyper-parameters *α* and *b* are shown in Table 4. It is noticed that the four choices of hyper-parameters yielded similar inferences for the posterior means of the fixed parameters. Minor differences, however, occur between the variance parameters for the nonlinear functions and the spatial effects suggesting the robustness of our choices. Thus, indicating that our model is less sensitive to the choice of hyper-parameters.

## Discussion

This study utilizes *geoadditive* modeling approach to develop a multivariate explanatory model for the risk of cholera. We utilize a Bayesian semi-parametric regression model to elucidate the probability of cholera infection in relation to associated risk factors, some identified from previous studies [11,12]. The *geoadditive* modeling approach is an extension of the GAM which allows the inclusion of both structured and unstructured spatial effects to account for possible unobserved factors and heterogeneity terms. To allow flexibility, the continuous covariates are modeled non-parametrically as nonlinear functions using P-splines with second-order random walk priors based, this based on contributions by Farhmeir and Lang [29,30] and Fahrmeir *et al.*[18]; while the categorical covariates are modeled as fixed effects. The spatially structured and unstructured effects are modeled using Markov random filed priors and zero mean Gaussian heterogeneity priors, respectively [31]. In this modeling approach, fully Bayesian inferences based on MCMC simulations are preferred because the functionals of the posterior can be easily computed, thereby easily quantifying the uncertainty in the estimated parameters [18].

The findings of the study show that the risk of cholera infection is high amongst inhabitants dwelling in slums. The risk of infection is also relatively high in densely populated communities. These relationships may exist because most communities with slummy settlers are densely populated. Although cholera is transmitted mainly through contaminated water or food, poor sanitary conditions in the environment enhance its transmission. The cholera *vibrios* can survive and multiply outside the human body and can spread rapidly where living conditions are overcrowded and where there is no safe disposal of solid waste, liquid waste, and human feces [3,4]. These conditions are mostly met in slummy and densely populated communities in Kumasi. Such high population density may necessarily result in shorter disease transmission paths, thus increasing the risk of cholera infection. Also, inhabitants living at slummy areas are generally poor, and face problems including access to potable water and sanitation. In many cases public utilities providers (e.g. water distribution) legally fail to serve these urban poor due to factors regarding land tenure system, technical and service regulations, and city development plans. Most slum settlements are also located at low lying areas susceptible to flooding. Unfavorable topography, soil, and hydro-geological conditions make it difficult to achieve and maintain high sanitation standards among such inhabitants [10].

The risk of cholera infection is observed to decrease with increasing distance from refuse dumps, inhabitants within 500 m away from the refuse dumps being the most vulnerable. This is consistent with the finding from previous studies when a quantitative assessment of critical distance discrimination on experimental buffer zones around refuse dumps showed that the optimum spatial discrimination of cholera occurs at 500 m way from refuse dumps [11]. Therefore, we hypothesize that refuse dumps located within 500 m away from inhabitants enhance the risk of cholera infection compared with those farther. The expected decreasing trend of *Chol*_(*R*)_ from $d_{\mathrm{dump}}\;\geq 500\;\text{m}$, however, is apparently grounds for strengthening the acceptance of this hypothesis. Collectively, the nonlinear effects of *d*_*dump*_ and *ρ*_*dump*_ on *Chol*_(*R*)_ suggest that cholera risk is relatively high amongst inhabitants who live in close proximity to refuse dumps, and where there are numerous refuse dumps. Due to the bad defecation practices of most inhabitants, the refuse dumps may contain high fecal matter. Surface drainage from such refuse dumps pollutes water sources with feces which when used perpetuates the transmission of cholera *vibrios*. If the runoff from waste dumps during heavy rains serve as the major pathway for fecal and bacterial contamination of rivers and streams, then it is likely that inhabitants living closer to water bodies where these runoffs flow into will have higher cholera prevalence than those who live farther. The observed decreasing cholera prevalence with increasing distance from potentially polluted surface water bodies (Figure 4), and the significant linear relationship between *d*_*dump*_ and *d*_*reser*_ (results from preliminary regression analysis: *β =* 0.67*, R*^*2*^ *=* 0.34*, p <*0.001) support this hypothesis.

Cholera is primarily driven by environmental and socio-economic factors [3,4]; prior knowledge indicates that geographically close communities will tend to have similar relative risks. Thus, indicating the existence of structured spatial variation in the relative risk. The structured spatial effects included in the model are surrogate measures of unobserved spatially correlated risk factors of cholera. The results show clear evidence of significant clustering of cholera, with higher cholera risk occurring at the central part (the Central Business District), and a lower risk occurring at the south-eastern part (the periphery) of Kumasi (Figure 5). These patterns clearly indicate possible unobserved risk factors of cholera, which may be global or local. For example, the increased risk at the central part of Kumasi may be an influence of high daily influx of traders and civil workers from other communities to the Central Business District. Such a high daily influx strain existing sanitation systems which consequently put people at increased risk of cholera. The dominancy of the unstructured spatial effects over the structured spatial effects indicates that the unobserved risk factors are more local than global. For instance, household socioeconomic characteristics may cause such local spatial variation. Therefore, this gives leads for further epidemiological research using additional information at household spatial scale within the study area.

Unlike classical modeling approaches, our methodological concept allows modeling flexibility which can reveal salient features of the continuous covariates. For instance, the utilization of only the linear model, Model 1, would have led to an invalid rejection of the significance of some important risk factors: density of refuse dumps, and proximity to potential cholera reservoirs. Such modeling approach is useful to establish a better epidemiological relationship that exists between the disease and the risk factors. Although the methodological concept is somewhat mathematically intensive, the availability of the public domain software, BayesX, provides opportunities for nonprogrammers to utilize these methods.

### Limitations of study

Data limitations have enforced this study to be undertaken within a single-scale framework; therefore, significance of scale effects has not been accounted for in this study. Consequently, possible biases induced by modifiable areal unit problem (MAUP) have been ignored. If data at different levels of spatial scales were available, possible bias of MAUP would be evaluated within a multi-scale analysis framework as exemplified in Odoi *et al.*[32]. Moreover, re-aggregating the data to another set of areal units could assess the possible bias of MAUP [33]. However, this is impossible due to the limited availability of higher resolution data and difficulties in assessing the ecological fallacy associated. In accordance with the general rule of practice, the study analyzed aggregated data using the smallest areal units for which data were available to ameliorate the effects of aggregation. Accordingly, statistical inferences in this study are emphasized on the group-level rather than the individual-level.

Also, our choice of neighborhood structure induces an assumption that all the inhabitants reside at the centroid of the communities. In reality, the communities have boundaries whereby their adjacency reflects the true nature of the spatial structure. Also, the maps of the spatial effects should be interpreted with caution as the spatial boundaries used are artificial (Thiessen polygons). Perhaps different spatial patterns may be visually observed if the true boundaries of the spatial units existed.

## Conclusion

This study applies a Bayesian semi-parametric modeling approach to develop an explanatory model of cholera. Such flexible modeling approaches allow joint analysis of nonlinear effects of continuous covariates, spatially structured variation, unstructured heterogeneity, and fixed effect covariates. Our model reveals that the risk of cholera infection is associated with slum settlements, high population density, proximity to and density of waste dumps, proximity to potentially polluted rivers and streams, as well as possible unobserved risk factors. The possible unobserved risk factors are shown by the distinct spatial patterns exhibited by the spatial covariates; suggesting the need for further epidemiological research. These findings should serve as novel information to help health planners and policy makers in making effective decisions about cholera control measures.

## Competing interests

The authors declare that they have no competing interests.

## Authors’ contributions

FBO carried out the research and drafted the manuscript. AAD and AS guided the research and reviewed the manuscript. All authors read and approved the final manuscript.

## Pre-publication history

The pre-publication history for this paper can be accessed here:

http://www.biomedcentral.com/1471-2288/12/118/prepub
